# Fracture of the Anatomical Neck of the Os Brachii, with Displacement of the Shaft

**Published:** 1859-07

**Authors:** Frederic D. Lente

**Affiliations:** Surgeon to the West Point Foundry


**NEW YORK MONTHLY REVIEW,**

AND

**BUFFALO MEDICAL JOURNAL.**

**VOL. 15.** JULY, 1859. **NO. 2.**

### ORIGINAL COMMUNICATIONS.

ART. I.—*Fracture at the Anatomical Neck of the Os Brachii, with displacement of the Shaft.* By FREDERIC D. LENTE, M.D., Surgeon to the West Point Foundry.

The subject of the above injury is Cadet M., of the West Point Military Academy. On the 22d of January, 1858, while going through with his equestrian exercises, his horse fell with him; his left shoulder was crushed between the horse and the ground. Swelling to a considerable extent ensued, rendering it difficult at the time to examine the part satisfactorily. It was treated by rest and evaporating lotions, but the tumefaction did not subside for a week or more. These particulars I learned from Dr. S. F. Moore, surgeon of the post, who, with Dr. Campbell, the assistant surgeon, examined the case. To-day, Feb. 5, I was requested by Dr. Moore to examine the shoulder. The tumefaction has now entirely subsided; and, as the patient is very spare, a very satisfactory examination can be made. Quite extensive passive motion at the joint, in all directions, is allowable, and without acute pain. There is a marked depression below the acromion, without flattening of the *deltoid*; and a marked prominence in front of the glenoid cavity, under the coracoid process, having the appearance of a partial dislocation of the head of the humerus forwards. Patient can move the fore-arm or the arm, but has little power over the latter. Upon rotating the shaft, and moving it freely backward and forward, bony crepitation, not exactly of the ordinary character, however, and yet quite distinct from cartilaginous, is readily perceived. But, upon pushing up the shaft towards the glenoid cavity, the crepitation disappears, although the contour of the shoulder is thereby in a great measure restored. Upon rotating the shaft, or upon the least motion of the arm by the patient himself, the prominence in front of the joint is seen and felt to move with it, and is evidently the upper extremity of the bone; but, instead of feeling smooth and globular, it has a distinct prominence, not sharp, but abrupt and slightly rounded, like the end of the coracoid process, for instance. Posterior to this prominence, and laying just under the glenoid cavity, is a smooth, globular body resembling the head of the bone. In rotating the shaft of the bone, this rotates also, but only to a moderate extent. By grasping this firmly, and at the same time, the anterior prominence, and rubbing them together, a bony crepitation, tolerably distinct, but not harsh like that of an ordinary fracture, is felt.

Our diagnosis is that, by the crushing force applied to the shoulder, the head of the Os Brachii was knocked off at the *anatomical neck*, and that the *tuberosities* were forced forward to the position already described. The head of the bone might also have been forced a little downwards at the time of the accident, or perhaps settled a little subsequently, so as to leave a depression below the acromion, as is not unfrequently the case after injuries about the joint. The deltoid is well developed for so thin a subject.

It was concluded to etherize the patient, make moderate extension of the humerus with appropriate counter-extension, and to endeavor to bring the upper end of the bone nearer its normal position. This was done, but nothing satisfactory resulted.

June 6th. Saw the patient to-day, through the courtesy of Dr. Moore. There has been such a degree of wasting of the tissues about the joint, that the condition of the parts can be almost as perfectly made out, as if they were dissected. The depression below the acromion is less marked, the deltoid is much wasted, but less so, Dr. Moore informs me, than it was a few weeks ago. Patient is slowly regaining the use of the arm.

April 8, 1859. It is now about fourteen months since the occurrence of the accident; for some months Mr. M. has been able to perform all the drill duty required of his class, satisfactorily, except infantry drill: he has trouble in bringing his musket quickly to the shoulder. I examined the shoulder to-day, with Dr. F. B. Richerson, of Virginia. Its appearance is almost precisely as when last described, with one exception: the arm is much shortened,—appearing to the eye at least a couple of inches shorter than the other. Upon measurement, the shortening is found to be full an inch and a half. The abrupt prominence before alluded to has a sharper outline, and is evidently the lesser tuberosity, being precisely in its position, and having precisely its feel. Upon rotating the shaft, the head of the bone follows its motions perfectly, and seems firmly attached; but, upon grasping the former securely, and rotating the shaft at the same time, slipping of one or the other is very perceptible, and indeed audible to a by-stander; when this is done the patient feels a dull pain, as if a sharp blow had been struck on the arm.

REMARKS. *Fracture at the anatomical neck of the Os Brachii* must, from its situation and the anatomical relations of the parts involved, be an extremely rare accident; and it is so regarded by all writers on the subject. Desault, who appears to have had a large experience in fractures about the shoulder-joint, says, "Examples of this nature (fracture at the anatomical neck) which occur in the annals of surgery, are too few to enable us to lay down any general principles of treatment."* There is also, as a general feature, so little precision in the language of surgeons when alluding to the extraordinary extra and intra-capsular fractures of the neck of the humerus, that is often impossible to interpret their experience satisfactorily. This is the case even with the editor of the Surgical Dictionary, who speaks of a fracture of the anatomical neck, and yet subsequently mentions the fact that it passed through a portion of the tuberosity; which makes the difference of a fracture within and one without the capsular ligament. And Vidal de Cassis, according to Dr. Smith, commits a still greater anatomical error in alluding to these fractures. Extremely few specimens have been preserved in the large museums. Sir Astley Cooper and Mr. Liston each met with a case of fracture of the anatomical neck,† where they had an opportunity of dissecting the parts; and in both instances, there was also dislocation of the head of the bone.

Dr. Smith, in his very excellent treatise on fracture in the vicinity of the joints, says that in fracture at the anatomical neck there is little or no displacement; because while the lower fragment is liable to be displaced inwards by the muscles constituting the folds of the axilla, this liability is counteracted by the muscles inserted into the greater tuberosity. At the same time he gives, as an important diagnostic mark of extra-capsular fracture, the fact that while, in rotating the shaft, its upper end can be plainly felt rolling under the fingers in concert with it, the head of the bone grasped firmly by the fingers of the other hand can be felt immovable in its natural position; and in the latter statement, most other writers agree with

---

* Desault's Surgery, Am. ed., p. 68.

† "Dislocations and Fractures." "Elements of Surgery."

him. Thus, the present case proves an important exception to this rule, if it be considered an extra-capsular fracture; for the head moved in concert with the shaft, thus producing considerable temporary embarrassment in the diagnosis of the injury. From the nature of the injury, being just such as might easily occasion such a result; from the feel of the upper extremity of the lower fragment, giving the exact impression, both to Dr. Moore and myself, that it was the lesser tuberosity rolling under the fingers, being also just in the position which that projection should occupy in such a condition of the parts; from the fact that the head moved in concert with the shaft, which is not observed in fracture at the epiphysis; and from the unusual amount of shortening,—I conclude that the fracture exists at the *anatomical neck*. The fact of the connection between the head and shaft being sufficient, notwithstanding the amount of displacement, to cause motion imparted to one to be transmitted to the other, indicates how the nutrition of the head is preserved in this accident, and even bony union consummated in cases of slight displacement; for it must have been through the medium of untorn shreds of periosteum and capsular ligament that the motion was communicated; and it is well known how great the vitality of the periosteum is, and how rapidly it gives rise to the formation of new bone, although its connection be very slight, and sometimes even when entirely detached from its natural connections.* The danger of the detached head acting as a foreign body in the joint and producing disorganization, is alluded to by most writers on the subject; but I am not aware of more than one recorded instance where such a result actually occurred. In fact, some of the instances recorded by surgical writers, comparatively few as they are, have been probably either fractures partly extra and partly intra-capsular, or of the *neck of the scapula*,—especially by those, who, like Erichsen and Smith, strange as it may appear, deny the possibility of this latter species of fracture.†

I can see no reason why displacement should not occur in fracture at the anatomical neck, if the force which produces the fracture continues to act on the upper end of the shaft, as it would most likely do in such an accident as that which produced the injury in this case. It would seem a much more remarkable occurrence that a fracture at the anatomical neck should occur, and the *head* at the same time thrust from its socket and

---

* Experiments on animals have lately demonstrated the fact that a strip of periosteum entirely detached from all connection with the bone, and thrust among the muscles in a distant part of the body, will not only maintain its vitality, but throw out bony matter rapidly.

† It is remarkable that Mr. Erichsen in his work on the "Science and Art of Surgery," should allude to Sir Astley Cooper as one of those who disbelieve in fracture of the neck of the scapula.

completely dislocated by the continuance of the force, as in the two cases referred to in this paper.

COLD SPRING, April 15, 1859.

---

[The following letter, detailing an interesting case, was handed to us by Dr. Hamilton. Ed.]

COLD SPRING, April 21, 1859.

MY DEAR SIR:—At your request I send you the notes of the case of fracture, alluded to in my last communication. I have recently had another very interesting fracture, that of the upper part of the Femur in a boy twelve years, by *muscular spasm* during a violent fit of *Epilepsy*, to which he has been subject from the age of fifteen months. What makes the case remarkable is the fact that the bones are apparently in a normal state, and also that the spasm was *tonic*, the thigh at the moment of fracture, being extremely flexed on the pelvis by a continuous pulling of the flexor muscles.

I remain yours truly,

To F. H. HAMILTON, M. D. FRED. L. LENTE.

